# Does experiencing driving-related challenges increase older adults’ likelihood of entering senior living communities?

**DOI:** 10.1093/geront/gnag089

**Published:** 2026-05-03

**Authors:** Sang-O Kim

**Affiliations:** Lewis Center for Regional Policy Studies, University of California Los Angeles (UCLA), 337 Charles E Young Drive, Los Angeles, California, 90095, United States

**Keywords:** Driving cessation, Senior living communities, Long-term care, Discrete-time event analysis

## Abstract

**Background and Objectives:**

By 2050, adults aged 65 and older will comprise roughly one-quarter of the United States population, heightening the importance of understanding factors that shape older adults’ residential choices. While most prefer to remain in familiar homes and communities, many relocate to senior-living communities such as retirement homes, board-and-care residences, or nursing homes. Among the factors that may shape these transitions, declining driving ability may be especially relevant in the US, where older adults remain heavily dependent on private vehicles to meet everyday needs, access healthcare, and maintain social connections. As driving becomes more difficult or impossible, older adults may face growing challenges to independent living. Yet the role of driving decline in residential transition has received limited empirical attention.

**Research Design and Methods:**

This study uses 9 waves of the National Health and Aging Trends Study (NHATS) from 2011 to 2019. A discrete-time event-history model was used to examine whether older adults’ driving status and experiences of driving-related challenges predict residential transitions to senior-living communities.

**Results:**

Non-driving status strongly predicts residential relocation. However, individuals who voluntarily self-regulate driving face relocation risks similar to those who have already ceased driving entirely. Among non-drivers, timing matters: older adults who recently ceased driving are substantially more likely to relocate than peers who stopped over a decade earlier, whose risk approximates that of current drivers.

**Discussion and Implications:**

These findings suggest that policy interventions aimed at expanding alternative transportation options may help older adults maintain independence and reduce the risk of entering senior-living communities.

This study investigates the relationship between driving status and the likelihood of older adults relocating from private residences to senior living communities in the United States. By 2050, it is projected that those aged 65 and older will reach a quarter of the nation’s population—or roughly 82 million people (Mather & Scommegna, 2024). With such a demographic shift, the question of older adults’ residential choice is becoming increasingly important. While most older adults express a strong preference to age in place in their current home and community, a substantial share do relocate to senior living communities (SLCs). In this paper, SLCs are broadly defined as residential settings that provide long-term housing and supportive services for older adults, including assistance with personal care, health-related needs, or transportation. The term, therefore, encompasses a diverse spectrum of facilities, including assisted living facilities, board-and-care homes, retirement villages, and nursing homes.

Driving status is one of the factors that may shape these residential decisions. Extensive studies have demonstrated that access to reliable transportation is essential for older adults’ ability to reach healthcare, grocery stores, community services, social networks, etc., which is required to maintain independence and well-being in later life (Adorno et al., 2018; Chihuri et al., 2016; Syed et al., 2013; Ward & Walsh, 2023). In the US, where most older adults rely heavily on a car to meet daily travel needs, the loss of driving ability may have a consequential impact on residential choice. Specifically, older adults who reduce or cease driving may be more likely to relocate to SLCs, where supportive services, including transportation, can help compensate for declining automobility.

In this context, based on the analysis of national longitudinal data on aging, this paper makes three contributions to the broad literature on driving and older adults’ housing choice. First, it provides more up-to-date empirical evidence on the relationship between driving status and moves from private residences to SLCs by quantifying differences in relocation likelihood between drivers and non-drivers. Second, by examining the residential outcomes of older adults who self-regulate their driving, the study highlights a need to treat driving cessation as a gradual process and that residential vulnerability may emerge before older adults stop driving completely. Third, by distinguishing recent versus long-term non-drivers and non-drivers experiencing transportation difficulty, the study shows that non-driving itself is not a uniform condition and that scholarship needs to go beyond a simple driver/non-driver binary in analyzing transportation-induced life outcomes of older adults.

The rest of the paper is organized as follows. The next section reviews prior research on older adults’ transitions to senior living communities and on the consequences of facing transportation-related challenges in later life. Then it draws these literatures together to identify knowledge gaps and present this study’s hypotheses. The following section outlines the data, measures, and empirical approach. The final sections present the findings and discuss their policy implications.

## Literature review

### Relocation to SLC: Trends, motivations, and transportation implications

While most older adults in the US express a strong preference to age in place, the number living in SLCs has steadily grown over time as well. The most recent federal data indicate that in 2022, there were more than 2.1 million residents living across roughly 32,200 SLCs nationwide (CDC, 2022). Demand is poised to rise sharply as demographic aging accelerates, with 20% of Americans projected to be of retirement age by 2030. Reflecting these trends, the industry leaders predict that the country will require approximately 800,000 additional units to meet the increasing housing needs of older adults by 2030, with the entire senior housing market expected to exceed $800 billion in value (Jones & Clem, 2025; NIC, 2024).

Research on older adults’ housing choices points to two broad explanations for relocation to SLCs. First, older adults are likely to relocate when their physical or cognitive capacities no longer align with the demands of their home environment—a finding commonly framed through Person Environment Fit theory (Choi, 2022; Lien et al., 2015; Safran-Norton, 2010). Other studies have portrayed relocation to SLC as an amenity-based move in which older adults are drawn by the services and advantages of SLCs that are not readily available in their current residential environment. Here, older adults are pulled by factors such as on-site healthcare, social opportunities, transportation services, recreational facilities, or even favorable climates (Bekhet et al., 2009; Chaulagain et al., 2021; Longino et al., 2002; Weeks et al., 2012).

In this context, several studies specifically show that transportation service availability is a significant pull factor among older adults considering relocation (Chaulagain et al., 2021; Choi, 2022). Most often, SLCs offer free shuttle services to shopping centers, grocery stores, and healthcare facilities, which allow older adults to access destinations that are essential to maintaining well-being and health without having to drive themselves. The national data show that almost 79% of SLCs provide such services (CDC, 2016). Furthermore, while less prevalent in the US literature, studies in European and Asian contexts suggest that proximity to public transit networks is often a significant pull factor as well (Croucher et al., 2003; Tyvimaa & Kemp, 2011). Taken together, these studies suggest that SLCs that provide transportation services may be especially attractive to older adults experiencing declines in driving ability.

Notwithstanding the importance of transportation as a pull factor, direct evidence on whether declining driving ability increases the likelihood of subsequent relocation to SLCs remains limited—the focus of this study. The closest parallel in the US literature can be found in the work of Freeman et al. (2006), which found that non-drivers had a higher hazard of entering long-term care facilities (including nursing homes, retirement homes, and assisted-living facilities) than drivers. However, that study was conducted more than two decades ago and relied on a sample from a small sample gathered from a rural Maryland town of roughly 40,000 residents, thereby limiting its contemporary relevance and geographic generalizability. Related studies provide indirect support by showing that older adults who relocate to environments with better transit access or closer proximity to important destinations often reduce their driving (Bekhet et al., 2009; Croucher et al., 2003; Schouten et al., 2022; Vivoda et al., 2017). However, they provide little insight into the reverse relationship: whether declining driving ability signals heightened risk of future relocation. The next section shows why this gap matters by examining older adults’ dependence on the automobile in the US.

### Role of driving in older adults’ life outcomes

Extensive studies show older adults’ travel demands remain high into later life. After retirement, baby boomers and older adults replace commuting trips with increased social and recreational trips to maintain their social networks (Cui et al., 2017; Siren & Haustein, 2016). Travel is also required for everyday needs such as grocery shopping, banking, and other routine errands, which do not disappear simply because one has retired (Musselwhite & Haddad, 2017; Schouten et al., 2022). Most significantly perhaps, older adults’ travel behavior is shaped by healthcare demands, such as attending routine check-ups or receiving specialized treatments, which become more frequent and unpredictable as they age (Adorno et al., 2018; MacLeod et al., 2015; Rosenbloom & Winsten-Bartlett, 2002; Syed et al., 2013). Furthermore, studies also show how driving can be a form of pleasure that brings hedonistic utility to older adults (Musselwhite & Haddad, 2017).

The majority of older adults rely on private vehicles to meet these everyday travel needs. Both car ownership rate and average vehicle miles traveled are significantly higher among American older adults than in other high-income countries such as Germany or the United Kingdom (Buehler et al., 2024). This pattern partly reflects the greater flexibility and convenience that private vehicles offer older adults. In the US context, however, such high levels of auto-dependence also reflect decades of transportation and land use policy that prioritized private cars over alternative modes (Blumenberg, 2016; Schouten et al., 2022). For instance, decades of under-investment have left many public transit systems inadequate in both quality and coverage (Fields et al., 2021; Ward & Walsh, 2023). The problem is worse in suburban and rural areas where a growing share of older adults live (Warner & Morken, 2013). Some communities provide dedicated senior transportation services, namely paratransit. However, these services are often criticized for their inefficiency and poor service quality (Kaufman et al., 2016). Declining physical capacity also limits older adults’ ability to use active modes such as walking and biking (Kim, 2011; Shirgaokar et al., 2020). As a result, many older adults rely on family or friends for transportation, but the availability of such informal support varies widely among individuals and often fluctuates over time (Savoie et al., 2024; Scharlach, 2017). Thus, despite having substantial and diverse travel needs, many older adults have few viable alternative transportation options to driving.

However, driving into old age is not something everyone can maintain. Declines in physical and cognitive capacity constrain older adults’ ability to drive and eventually lead to driving cessation (Chihuri et al., 2016; Liddle et al., 2008). Even those retaining car access confront restrictions such as vision impairments, heightened injury risk, and safety concerns, which constrain when and where they drive (Ang et al., 2019; Musselwhite & Shergold, 2012). Financial pressures compound these challenges. With loss of stable income after retirement, costs of car ownership (i.e., insurance, gas, repairs, etc.) can themselves become financially burdensome (Adorno et al., 2018; Siren & Haustein, 2016). These issues disproportionately affect low-income older adults, whose numbers are rising in the US (Loukaitou-Sideris et al., 2018).

Not surprisingly, driving cessation is associated with a wide range of adverse outcomes. Driving cessation is associated with reduced independence, lower self-esteem, and, in some studies, nearly double the risk of depression and dementia among older adults (Chihuri et al., 2016; Liddle et al., 2024; Musselwhite & Haddad, 2017). Older adults who stop driving also participate in fewer out-of-home activities and therefore face greater risks of social isolation (Adorno et al., 2018; Curl et al., 2014; Loukaitou-Sideris et al., 2018). In addition, the loss of driving ability can make it more difficult to access nutritious food and obtain timely healthcare, both of which are closely tied to health outcomes (Syed et al., 2013; Valliant et al., 2022). These adverse effects are strongest in the period immediately following driving cessation and often diminish over time, suggesting a process of adaptation among older adults (Liddle et al., 2024).

More recently, a growing body of scholarship has emphasized the need to treat driving cessation as a gradual and negotiated process, and to recognize older adults who self-regulate driving as their own category (Ang et al., 2019; Donorfio et al., 2009; Liddle et al., 2008; Sawada et al., 2025). These older adults continue to drive, but limit their driving under certain conditions, such as at night, alone, or in bad weather. Scholars point out that this period is often marked by psychological and material struggle in which older adults grapple with questions of independence and self-worth, while also beginning to search for new ways of coping with the next stage of life without a car and smaller activity space. This call also resonates with a broader strand of transportation research that urges scholars to move beyond a simple driver versus non-driver distinction and instead examine heterogeneity in each group and associated life outcomes (Curl et al., 2018; Johnson et al., 2010). Yet contemporary evidence remains limited to how these different stages of driving are associated with a different likelihood of relocation to SLCs.

### 2.3 Research gaps and hypotheses

Taken together, the literature suggests that older adults experiencing declines in driving ability may be more likely to relocate to SLCs, many of which help older adults to reduce dependence on private vehicles through transportation services and on-site amenities. However, contemporary empirical evidence on whether declining driving ability increases the likelihood of subsequent relocation to SLCs remains limited. Moreover, there is almost no research examining whether relocation risk differs across stages of driving decline, such as self-regulating drivers, recent non-drivers, or non-drivers who report acute transportation challenges. To address these gaps, this study uses national longitudinal data to examine how variation in driving ability is associated with the likelihood of transition to SLCs. Specifically, the study tests the following four hypotheses:

(H1) Non-driving older adults have a higher likelihood of transitioning to SLCs than driving older adults;

(H2) Older adults who still drive but report constrained driving ability have a higher likelihood of transitioning than fully able drivers;

(H3) Non-driving older adults experiencing transportation difficulty have a higher likelihood of transitioning than non-drivers with no reported difficulty;

(H4) The likelihood of transitioning to SLCs decreases as the duration of non-driving increases.

The next section provides an overview of the method and data used for this study.

## Method and data

### Data description

This study draws on data from the National Health and Aging Trends Study (NHATS), a longitudinal panel survey that has tracked the life course of Medicare beneficiaries aged 65 and older in the US since 2011. Through annual in-person interviews, NHATS collects detailed information on socio-demographic characteristics, health conditions, functional status, mobility and transportation, and housing status of sampled older adults. For respondents living in institutional settings, additional facility questionnaires are conducted for staff members to supplement the main survey. For this study, data are limited to the years between 2011 and 2019 to control the disruptive impact of COVID-19. Hence, the unit of analysis for this study is the individual-year observation.

NHATS classifies types of respondents’ residences into five categories: (1) Private Residence; (2) Group home, board & care, or supervised housing; (3) Assisted-living or continuing care retirement community; (4) nursing home; (5) Other. Based on this categorization, two types of residential transition flags were created—one for those who moved from a private residential setting (1) to a nursing home (4), and another for those who moved from a private residential setting (1) to a non-nursing home SLCs (2 and 3) (henceforth residential care communities). This distinction follows research in gerontology highlighting differences in health, functional status, and socioeconomic background between nursing home residents and those in residential care communities (Lum et al., 2015; Toth et al., 2020; Wang et al., 2023). Since our focus is relocation from private residences, individuals who were already living in nursing homes or residential care in the baseline year were excluded.

After the initial data cleaning, 10,953 NHATS respondents were identified as living in a private residence at Wave 1. By the end of the study period (i.e., Wave 9), most older adults (about 73%; *n* = 8,066) still remained in a private residence. However, 7.3% of the sample (*n* = 794) moved from a private residence into either a residential care community (*n* = 468) or a nursing home (*n* = 326). In addition, 2,093 respondents who were living in a private residence at Wave 1 were no longer observed by Wave 9 because they either died or were otherwise lost to follow-up. Given the focus of this study, the subsequent discrete-time event analysis centers on those 794 older adults who made a residential move into either a residential care community or a nursing home.

### Analytical model set up

**Figure 1:**
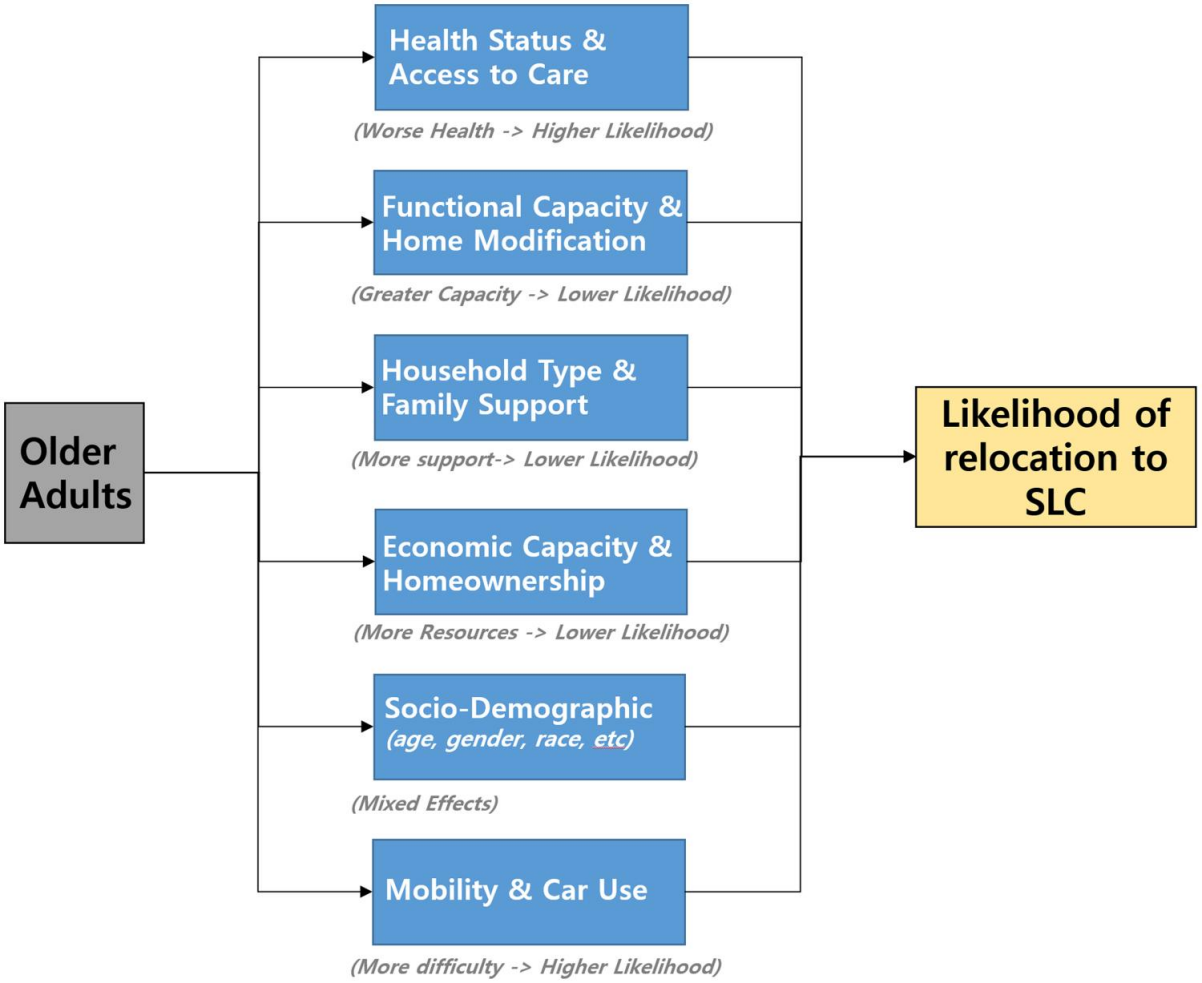
This figure presents the conceptual framework guiding this paper’s statistical analysis of older adults’ likelihood of relocating to senior living communities. It summarizes key domains and expected directions of association, including health and access to care, functional capacity and home modification, household and family support, economic resources and homeownership, socio-demographics, and mobility and car use.

To test the four hypotheses presented above, four transportation variables were constructed. First, older adults in NHATS were classified as drivers or non-drivers. Among drivers, those self-regulating driving were identified based on NHATS questions indicating whether older adults self-restrict driving at night, on highways, alone, or in bad weather. Among non-drivers, those who reported experiencing significant transportation-related barriers to accessing places were identified using questions on whether transportation prevented them from engaging in social activities such as meeting family or friends, participating in community events, or engaging in leisure activities. Non-drivers were further categorized as recent or long-term non-drivers based on duration since driving cessation. These driving status variables were analyzed alongside common predictors of residential transitions identified in prior studies. Figure 1 presents the conceptual framework, and Supplementary Appendix 1 describes all variables in detail.

The study used two models. To test hypotheses H1, H2, and H3, a discrete-time event-history model was used. This approach evaluates the discrete-time hazard function and estimates the likelihood that an older adult relocates to an SLC at a given wave, conditional on not having transitioned in earlier waves. This model allows the effects of driving status and other covariates on the timing of transition to be quantified. Respondents were right-censored at death, loss to follow-up, or the end of Wave 9 if they remained at a private residence. Model fit was evaluated using plots of the baseline hazard and likelihood-ratio tests. Standard errors are clustered at the respondent level. As a robustness check, the study implemented a respondent-level block bootstrap (1,000 replicates) and examined the sensitivity of model outputs to alternative specifications of key variables relating to driving status, household structure, and self-rated health. Throughout these checks, the main results remained the same. For H4 specifically, a simple binary logit model was used. This was because the duration of non-driving among older adults in NHATS often exceeded the nine-year observation window. As such, the data had to be collapsed to a single dimension to test the effects of covariates, including non-driving duration, on chances of relocation. All data management and statistical analyses were conducted in R version 4.5.1 (R Core Team, 2025).

## Findings

### Relationship between driving status and chances of relocation

Table 1 summarizes how a variety of factors shape the risk of moving from private residences to a residential care community. Living arrangements and household resources emerge as particularly significant factors: older adults living with family had about 1.5 times lower odds of relocation than those living alone, while those living with non-family others had odds that were essentially near zero, suggesting that the support from co-residents buffers against pressures that lead to institutional placement. Older adults who required assistance with meal preparation were a particularly strong signal of transition risk as they reported roughly nine-times higher odds of relocating than peers.

**Table 1:** Discrete time-event model for those moved to residential care communities.

Variable	Estimate	Std. error	*Z*-value	*p*-value
(Intercept)	−10.88	0.72	−15.16	<.001
**Age (Ref: 65–74)**				
Age 75–84	0.69	0.23	3.01	.003
Age 85+	1.38	0.24	5.84	<.001
**Gender (male)**	−0.52	0.18	−2.90	.004
**Race/ethnicity (Ref: non-Hispanic White)**				
African-American	−1.01	0.24	−4.18	<.001
Hispanic	−1.86	0.49	−3.81	<.001
Other race	−0.58	0.41	−1.42	.157
**Metropolitan location (non-metro)**	−0.47	0.22	−2.15	.031
**Economic asset**				
Receiving welfare	−1.18	0.25	−4.76	<.001
Household income	2.2e-8	1.1e-7	0.20	.839
Homeownership (renter)	3.03	0.32	14.44	<.001
**Community trust (Ref: low trust)**				
Medium	0.16	0.24	0.67	.501
High	0.84	0.22	3.88	<.001
**Household structure (Ref: living alone)**				
Live with family	−0.65	0.19	−3.44	<.001
Living with non- family others	−4.43	0.60	−7.40	<.001
**Self-rated health (Ref: poor health)**				
Fair	0.42	0.42	1.01	.314
Good	0.71	0.40	1.76	.078
Very good	0.42	0.42	1.01	.311
Excellent	0.60	0.46	1.31	.192
**Using telehealth**	0.29	0.24	1.22	.224
**Home modification (Ref: no modification)**				
Few modification	1.77	0.34	5.17	<.001
Moderate modification	1.88	0.38	4.96	<.001
Many modification	2.61	0.56	4.69	<.001
**Family provided support**				
Financial help	−0.10	0.25	−0.40	.690
Meal prep help	2.17	0.17	12.70	<.001
Transportation help	−0.03	0.19	−0.18	.860
**Driver status (non-driver)**	−0.93	0.20	4.66	<.001
Null deviance	2994.6 on 37568 df			
Residual deviance	1432.6 on 37538 df			
AIC	1498.6			

*Note.* This table reports the associations between older adults’ socio-demographic characteristics, economic and housing resources, community and household context, health-related factors, supports, home modifications, and driving status with the likelihood of moving from a private residence into a residential care community over the study period. Estimates are log-odds from a discrete-time logistic regression predicting residential care entry between NHATS waves. Baseline hazard is captured by wave dummies (not shown).

Home environment factors also showed strong associations. The largest effect was observed for housing tenure. Renters showed nearly 20 times higher odds of entering residential care compared to homeowners. In addition, home modifications emerged as a powerful marker of transition risk. Compared with older adults who reported no senior-friendly modifications, those with a few or moderate modifications had roughly six to seven times higher odds of relocation, and those with many modifications had more than ten times higher odds.

Among demographic indicators, gender and race were also significant. Men had odds of relocating that were about 1.7-times lower than women. African-American and Hispanic older adults had odds of residential relocation that were about three times lower and six times lower, respectively, compared with non-Hispanic White counterparts. In addition, age also displayed a clear gradient: adults aged 75–84 had odds of relocation that were almost two times higher than those aged 65–74, and those 85+ had odds more than four times higher. Older adults in non-metropolitan areas showed roughly 1.7 times lower odds of entering residential care than their peers in metropolitan regions. Finally, having a high level of trust in the surrounding community was associated with higher odds of relocation, whereas medium trust was not significant.

Driving-related factors were also significant predictors of relocation. After statistically controlling for age, health, and household supports, non-drivers still had about three times the higher odds of moving into residential care than active drivers. This suggests that the loss of automobility can hasten transitions out of the home. By contrast, receiving help with driving from family members was not a significant predictor. This likely reflects overlap with broader measures of disability and support needs (e.g., assistance with meals, age, overall health), so that transportation help contributes little unique information about relocation risk after those are accounted for.

Table 2 presents predictors of moving from home into a nursing home. Similar to the previous model, meal-preparation help was the strongest predictor. Driving status was also one of the clearest signals of risk, as respondents who no longer drove had odds of nursing-home entry about 12 times higher than drivers. Being a renter was also associated with elevated odds. Home modification status showed mixed associations relative to no modification. While living in a home with few modifications was associated with higher odds of transition, other categories did not show a consistent gradient. On the other hand, co-residing with family member(s) offered some signs of reducing the risk of transition. Demographic factors—age, gender, and race/ethnicity—were not significant once functional status and resources were taken into account. Self-rated health, community trust, and use of telemedicine also showed no independent association. Overall, the nursing-home model largely echoes findings for residential care: limitations in daily living (i.e., meal prep), lack of housing security (i.e., renter status), and loss of independent driving remain key indicators of relocation.

**Table 2:** Discrete time-event model for those moved to nursing home.

Variable	Estimate	Std. error	*Z*-value	*p*-value
(Intercept)	−30.14	3531.00	−0.01	.993
**Age (Ref: 65–74)**				
Age 75**–**84	0.80	1.18	0.68	.498
Age 85+	−0.12	1.25	−0.09	.926
**Gender (male)**	−0.34	0.67	−0.51	.614
**Race/ethnicity (Ref: non-Hispanic White)**				
African-American	−1.95	1.16	−1.67	.095
Hispanic	−1.38	1.18	−1.17	.241
Other race	−19.08	748.00	−0.01	.998
**Metropolitan location (non-metro)**	−0.07	0.71	−0.10	.924
**Economic asset**				
Receiving welfare	−1.01	0.90	−1.12	.262
Household income	−4.3e-7	6.8e-6	−0.06	.950
Homeownership (renter)	2.84	1.10	2.59	.014
**Community trust (Ref: low trust)**				
Medium	−0.31	0.86	−0.36	.717
High	−0.07	0.79	−0.09	.932
**Household structure (Ref: living alone)**				
Live with family	−1.57	0.86	−1.82	.068
Living with non- family others	−20.03	309.00	−0.01	.003
**Self-rated health (Ref: poor health)**				
Fair	0.45	1.21	0.37	.711
Good	0.39	1.39	0.33	.740
Very good	−0.01	1.34	−0.01	.995
Excellent	0.05	1.60	0.03	.977
**Using telehealth**	−17.18	342.00	−0.01	.966
**Home modification (Ref: no modification)**				
Few modification	8.43	0.53	15.0	<.001
Moderate modification	−1.31	0.63	−2.06	.039
Many modification	−0.76	0.88	−0.85	.394
**Family provided support**				
Financial help	0.54	0.75	0.67	.394
Meal prep help	4.02	1.09	3.70	<.001
Transportation help	0.17	0.83	0.21	.836
**Driver status (non-driver)**	2.53	1.23	2.07	.039
Null deviance	264.8 on 37568 df			
Residual deviance	112.0 on 37536 df			
AIC	178			

*Note.* This table reports the associations between older adults’ socio-demographic characteristics, economic and housing resources, community and household context, health-related factors, supports, home modifications, and driving status with the likelihood of moving from a private residence into a nursing home over the study period. Estimates are log-odds from a discrete-time logistic regression predicting residential care entry between NHATS waves. Baseline hazard is captured by wave dummies (not shown).

Together, these findings provide broad support for the first hypothesis (H1), indicating that non-drivers, who are likely to face more challenges in accessing destinations and thus have more unmet travel needs, have higher odds of making a residential transition than active drivers who have access to personal vehicles.

### Internal differences within the driver and non-driver groups

In addition, discrete-time event models were estimated separately for drivers and non-drivers, using the same covariates as the main analyses. These models assessed whether additional barriers to daily mobility influence the likelihood of relocation within each group. Specifically, they tested H2 and H3, which relate to whether drivers who self-regulate when or how they drive (H2) and non-drivers who report acute challenges with reaching destinations (H3) face a heightened risk of moving from home into SLCs encompassing both residential care communities or nursing homes.

**Figure 2:**
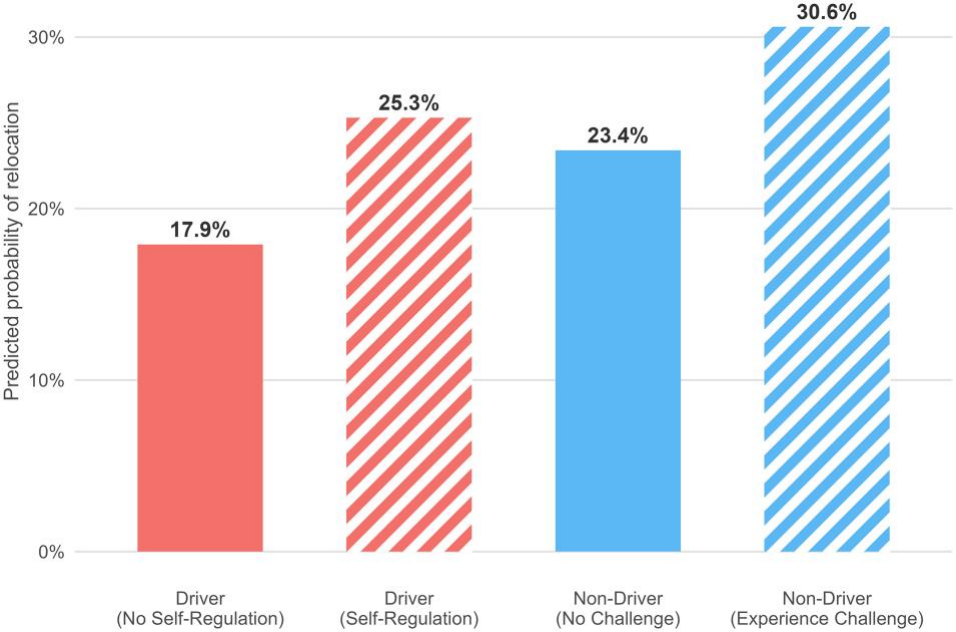
This figure presents predicted probabilities of relocation from the discrete-time event models, stratified by driving status and transportation challenges. Among drivers with no self-reported restrictions, the estimated probability of moving to residential or nursing care is about 17.9%. This rises to 25.3% for drivers who report self-regulated driving such as avoiding night driving, driving alone, highways, or bad weather conditions. Among non-drivers, the baseline probability is 23.4% for those reporting no major transportation difficulties, increasing to 30.6% for those who report added challenges reaching family and friends or social and leisure destinations. Overall, the results suggest that mobility barriers meaningfully increase the risk of relocation for both groups and support H2 and H3.

Figure 2 presents predicted probabilities of relocation from the discrete-time event models, stratified by heterogeneity in driving ability. Among drivers with no restrictions, the estimated probability of moving to residential or nursing care is about 17.9%. This probability rises to 25.3% for drivers who report self-regulation in terms of when or how they drive by avoiding driving at night, alone, using the highway or in bad weather. For non-drivers, the baseline probability is 23.4% when they report no major transportation difficulties. Note that this figure is higher than active drivers but lower than self-regulating drivers. However, the figure climbs to 30.6% for those non-drivers who experience additional challenges meeting family & friends or accessing social & leisure destinations. The findings, therefore, provide support for both Hypothesis 2 and Hypothesis 3 in that drivers who are engaged in self-regulation (H2) and non-drivers who experienced significant transportation-induced barriers. (H3) face a heightened risk of relocation than their respective reference groups (i.e., active drivers & non-drivers).

### How driving cessation duration shapes relocation likelihood

The final analysis examined whether the duration of non-driving shaped the likelihood of relocation, specifically whether older adults who had stopped driving recently differed in their relocation likelihood from those who had been non-drivers for a longer period (H4). The NHATS data ask non-driving respondents how many years ago they stopped driving in their initial intake surveys. Oftentimes, this duration of non-driving went beyond the scope of the panel dataset (Waves 1–9 covering nine years). As a result, this analysis used a logit model to predict how differing driving durations influence the probability of relocation. Specifically, non-driving duration was divided into three-year increments (i.e., 1–3 years, 4–6 years, 12 years, or more). To run the logit model, values of other independent variables were taken from the last observed wave in which the respondent was living in a private residence: for those who relocated, measures from the wave immediately preceding relocation were used, while for those who did not relocate, measures from Wave 9 (the final wave) were selected.

**Figure 3:**
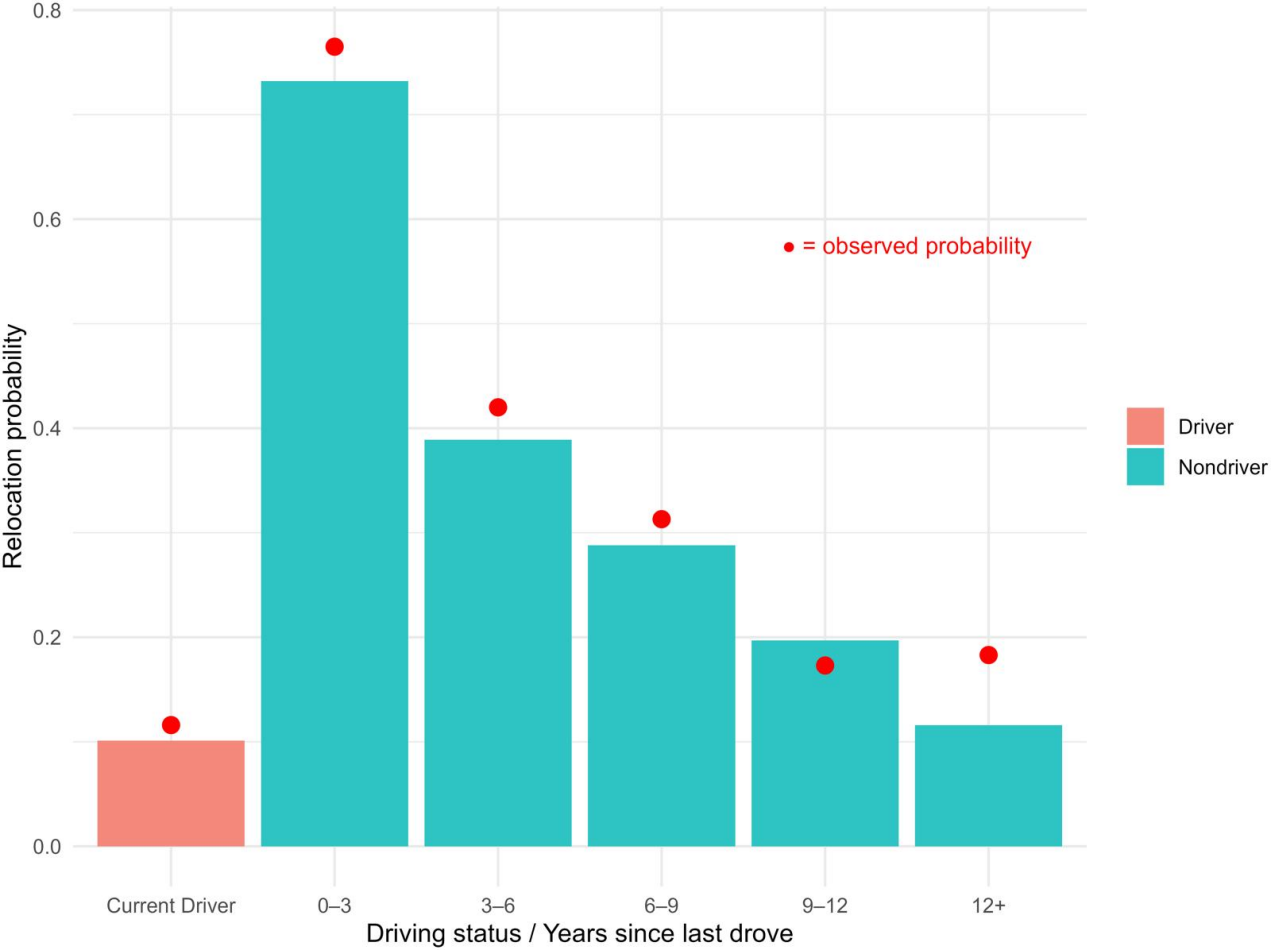
This figure presents predicted and observed probabilities of relocation by driving status and time since driving cessation. Current drivers show the lowest predicted risk, about 10%, while non-drivers face the highest risk within 1 to 3 years of stopping driving, about 73%. Predicted risk then declines steadily with longer durations of non-driving, approaching the level of drivers, consistent with Hypothesis 4.

Figure 3 provides the result from this logit analysis. The red bar on the left indicates that current drivers have the lowest predicted probability of relocation (i.e., 10%) with observed rates (red dots) closely matching model predictions. Among non-drivers, the likelihood of relocation is highest among those who gave up driving within the past 1–3 years, with a predicted probability of roughly 73%. As the time since driving cessation increases, the predicted risk declines steadily—from about 39% for those who stopped 3–6 years ago, 20% for 9–12 years, and down to about 12% for people who stopped more than 12 years ago. This pattern suggests that relocation risk peaks soon after driving cessation and diminishes as non-driving older adults make long-term adjustments to a life without a car, to a level that is comparable to drivers. Overall, these findings provide support for Hypothesis 4, indicating that the longer the duration of non-driving, the lower the likelihood of residential transition.

## Discussion

This study examined how variation in driving ability is associated with older adults’ likelihood of transitioning from private residences to SLCs. The findings, first and foremost, demonstrate that non-drivers have a substantially higher likelihood of residential transition than active drivers. This result is broadly consistent with earlier work by Freeman et al. (2006), while extending that work through contemporary longitudinal national data. Substantively, it provides evidence to support the argument that relocation to SLCs may function as a compensatory response to the loss of automobility required for aging in place for older adults. More importantly, perhaps the study moves beyond the simple driver/non-driver distinction and identifies three additional groups with an elevated likelihood of relocation.

First, older adults who still drive but report constrained driving ability, or self-regulating drivers, also appear to face an elevated likelihood of residential transition. This suggests that the residential implications of mobility decline may begin well before complete driving cessation. Prior literature has emphasized that driving cessation is often preceded by a phase of self-regulation during which older adults go through psychological reassessment of independence, self-worth, and social connection, as well as practical lifestyle adjustments in preparation for a life without automobility (Liddle et al., 2008; Sawada et al., 2025). This finding contributes to that body of literature by providing empirical evidence that this transitional phase may overlap with residential transition as well. In other words, older adults are likely to make changes to their housing arrangements while they are still driving, but are already experiencing clear signs that continued automobility is becoming difficult to sustain. In doing so, it adds nuances to the discourse that portrays relocation as a response or outcome to the driving cessation (Chihuri et al., 2016; Freeman et al., 2006). It also shed a new light on the transportation studies, which found reduced driving among older adults who relocated to denser, more walkable, or transit-rich neighborhoods (Schouten et al., 2022; Vivoda et al., 2017). Instead, it establishes a more complex picture where driving reduction has already been happening for older adults before they made residential relocation.

Second, the likelihood of relocation appears to be highest among older adults in the immediate post-cessation period, that is, those who stopped driving relatively recently. This pattern is consistent with prior research showing that the adverse effects of driving cessation are often strongest in the period immediately following the loss of driving (Liddle et al., 2024). In contrast, the likelihood of relocation appears to decline as the duration of non-driving increases, eventually becoming similar to that of active drivers. This is an intriguing result, given that transportation research consistently demonstrates how patterns of the built environment in the US place people without cars at a clear disadvantage in reaching destinations, and how older adults rarely have alternative modes of travel at their disposal (i.e., public transit, walking, or cycling) (Adorno et al., 2018; Blumenberg, 2016; Kim, 2011; Loukaitou-Sideris et al., 2018). These long-term non-drivers could be an important group for both policymaking and research purposes, as they illustrate the feasibility of aging in place without a private vehicle. In other words, understanding how these older adults maintain access to daily needs from their home without having to rely on car ownership could guide how planners, transit agencies, and community organizations should design environments that support healthy aging without dependence on automobiles.

Third, among non-drivers, those who report continuing transportation difficulties also face a higher likelihood of transition than those who do not. This suggests that non-driving alone is not what matters most. Rather, what appears consequential is whether older adults are able to meet everyday travel needs after cessation through alternative arrangements, such as informal support, community transportation, or other locally available resources. In that sense, the findings reinforce the idea that the consequences of non-driving are uneven. Some older adults appear able to remain in place after cessation, while others face persistent unmet travel needs that may make relocation to a more supportive residential environment more likely.

Taken together, these findings highlight the limitations of relying on a simple driver/non-driver distinction in research on later-life mobility and residential change. In both gerontology and transportation studies, older adults who no longer drive are often treated as a uniquely vulnerable group, and much of the literature has focused on documenting the disadvantages and negative life outcomes associated with driving cessation. Policy responses have largely followed the same logic by concentrating attention and resources on those who have already stopped driving. The present findings suggest that this framework is too narrow. It risks overlooking older adults who technically maintain car ownership and a license, but whose actual driving has already become constrained enough to reduce their activity space and increase their vulnerability. At the same time, the findings show that non-drivers themselves are far from a uniform category. The residential consequences of non-driving vary meaningfully according to how long older adults have been without a car and whether they are able to meet everyday travel needs through alternative means.

The study therefore supports a more heterogeneous and process-based understanding of how changing driving ability shapes later-life outcomes. Rather than a discrete shift from driver to non-driver, driving decline is better understood as a dynamic continuum that includes gradual constraint, cessation, and differing degrees of post-cessation adaptation. From this perspective, vulnerability may begin to emerge well before complete driving cessation and may evolve differently across older adults after cessation. In this sense, the paper contributes to gerontology literature by reinforcing calls to treat driving cessation as a negotiated life transition rather than a discrete event (Ang et al., 2019; Donorfio et al., 2009; Liddle et al., 2008; Sawada et al., 2025). In a similar vein, it contributes to transportation and urban planning scholarship by showing the limits of conventional measures, namely car ownership, for capturing how transportation disadvantage shapes life outcomes (Curl et al., 2018; Johnson et al., 2010).

## Policy implications

Likely, transportation interventions alone cannot prevent transitions to SLCs. Nevertheless, the findings suggest that improving alternative transportation options may help ease some of the challenges related to accessing destinations and unmet travel needs that contribute to relocation pressures among older adults with limited automobile access. This possibility matters not only because many older adults prefer to age in place themselves, but also because transitions to more supportive residential settings can incur substantial public expenditures (e.g., Medicaid Long-Term Care) and administrative burdens (e.g., social service agencies) (Brewster et al., 2020; CMS, 2024). As such, delaying, if not preventing, entry to SLC could yield meaningful individual well-being and societal benefits.

At the community level, the priority should be to maintain and expand alternative transportation services. The results for older adults who recently stopped driving are especially consistent with the idea that relocation pressure rises when car access is lost, and there is no viable alternative in a community. Across the nation, paratransit services that offer free & low-cost ADA-accommodation rides to older adults remain the most popular alternative. Many older adults enrolled in Medicaid also have non-emergency transportation services (NEMT) to meet healthcare travel needs. Yet both are widely constrained by poor service quality, difficulty of use, and uneven availability (Kaufman et al., 2016; MacLeod et al., 2015). Most significantly, most of these services require advanced booking, which means that they cannot accommodate spontaneous trips (Fields et al., 2021). To address these limitations and ensure paratransit and NEMT serve as a viable alternative, communities should actively seek to embrace ride-hail technology. To address these limitations, communities should explore the integration of ride-hailing technology into paratransit and NEMT systems. In principle, such intelligent paratransit could respond to demand more flexibly and efficiently, creating benefits for both agencies and older adults (Adorno et al., 2018; Fields et al., 2021; Kaufman et al., 2016). Communities should therefore assess latent demand and undertake careful cost-benefit analyses of these technological investments.

More fundamentally, a broader planning commitment is needed to address the continued marginalization of transportation domains within the policy realm on aging. In other words, as long as transportation remains underrecognized in aging policymaking, mobility-related investments are likely to be deferred, further intensifying the transportation-related risk of an aging society. Several scholars, therefore, argue that one of the most important policy interventions is to build a broad coalition of aging advocates that will create political capital to reshape local policy priorities and governance arrangements (Adorno et al., 2018; Warner & Morken, 2013). In this context, Area Agencies on Aging are often identified as being especially well-positioned to play a leadership role in such coalition building by disseminating knowledge about the costs of transportation inequity, empowering older adults, and fostering cross-sector collaboration (Cherlin et al., 2023; Gallo et al., 2022). To fulfill this role effectively, however, AAAs would require greater investment and stronger institutional capacity.

## Conclusion

As population aging accelerates, research on older adults’ housing transitions increasingly needs to account for mobility as a central part of residential wellbeing. This study addressed that issue by examining how variation in driving ability is associated with the likelihood of transition from a private residence to a senior living community. Overall, the findings suggest that declining driving ability is associated with heightened likelihood of transition to SLCs, but also that this relationship is more nuanced than a simple comparison between drivers and non-drivers would imply. In particular, the results point to self-regulating drivers and recent non-drivers as groups facing especially elevated residential vulnerability. In this sense, the findings reframe mobility decline as more than a problem of transportation disadvantage or accessibility, showing that it can also undermine wellbeing more broadly by shaping residential trajectories themselves.

This study is not without limitations. The analysis relies on observed residential transitions and cannot directly capture older adults’ underlying housing preferences. As a result, it cannot distinguish cleanly between moves that were reluctant responses to declining mobility and moves that were already desired for other reasons. Second, the study assumes that latent unmet travel demand generated by declining driving ability is an important mechanism linking mobility decline to relocation. However, different unmet travel needs are unlikely to have equal impact on older adults. Missing a healthcare trip, for instance, may have different implications from missing trips for grocery shopping. These dimensions no doubt play a critical role in residential decision-making and the role of transportation within. Future research would therefore benefit from qualitative approaches that engage older adults directly and collect data on residential preferences, perceived accessibility, and latent travel demands that are difficult to observe in revealed data alone. Qualitative research would complement the present findings by illuminating dimensions of decision-making and lived experience that are not easily captured in quantitative data, thereby offering a fuller understanding of how changes in driving ability shape residential trajectories in later life.

## Supplementary Material

gnag089_Supplementary_Data

## Data Availability

This research used publicly available data from the National Health and Aging Trends Study (NHATS). NHATS is produced and distributed by www.nhats.org with funding from the National Institute on Aging and Office of Behavioral and Social Sciences Research (OBSSR) (grant number U01AG032947). No original data were collected for this study. The NHATS public-use files are available to qualified researchers through the NHATS website and should be cited in the reference list in accordance with data citation standards. The analytic approach is replicable. However, the analytic code and related materials are not currently publicly archived because additional planned analyses using these files are ongoing. The code may be made available by the corresponding author upon reasonable request. The study was not pre-registered. This research is a secondary analysis of de-identified, publicly available data, and no additional institutional ethics approval was required for the analyses reported here.
