# Supplementary material for: Does experiencing driving-related challenges increase older adults’ likelihood of entering senior living communities?
Source: Gerontologist. 2026 May 3;66(7):gnag089. doi: 10.1093/geront/gnag089 (PMC13260861; doi:10.1093/geront/gnag089)
Supplement: gnag089_Supplementary_Data [file gnag089_supplementary_data.zip › [Revision]SangOKim_supplementary[April2026].pdf]

Does experiencing driving related challenges increase older adults' likelihood of entering senior living communities?

Sang-O Kim

Postdoctoral scholar, Lewis Center for Regional Policy Studies, University of California Los Angeles

Contact:

sangokim@ucla.edu

Supplementary Material

Appendix 1: Overview of Predictors Used in Analysis

Variable Group / Name	Type	Description / Categories
**Group 1: Socio-Demographic**		
Gender	Fixed	Female / Male
Age	Time-varying	65~74 / 75~84 / 85 and over
Race	Fixed	Non-Hispanic White, African American, Hispanic Other & Mixed Race
Metropolitan Location	Time-varying	Living in a census-designated metropolitan area
Community Trust	Time-varying	High Trust / Medium Trust / Low Trust
**Group 2: Economic Capacity**		
Total Household Income	Time-varying	Continuous numeric variable
Home Ownership	Time-varying	Homeowner / Renter
Welfare Benefits	Time-varying	YES if a respondent indicated receiving any one of the following programs: Food Stamp, HUD Energy Assistance, or Supplementary Security Income
**Group 3: Health Status & Access to Care**		
Self-Rated Health Status	Time-varying	Excellent, Very Good, Good, Fair, Poor
Telehealth Use	Time-varying	YES if a respondent indicated using either telehealth or telemedicine services regularly
**Group 4: Home Environment & Informal Support**		
Household Type	Time-varying	Living Alone / Living with family / Living with other non-family individuals
Home Modification	Time-varying	High modification, Moderate Modification or Little Modification based on the availability of the following six(6) modifications at the respondent's residence: stair lift, grab bar in shower, grab bar around toilet, raised toilet, ramp, a seat for the shower or tub
Family provide financial help	Time-varying	Yes / No
Family provide help with meal prep	Time-varying	Yes / No
Family provide help with transportation	Time-varying	Yes / No
**Group 5: Mobility & Driving**		
Driving Status	Time-varying	Whether a respondent operated a motor vehicle

Restrict Driving<br>(for drivers only)	Time-varying	Respondent does not feel comfortable driving alone / when dark / on highway / bad weather
Experience accessibility challenges<br>(for non-drivers only)	Time Varying	Transportation issues prevented visiting friend & family / attending community events / going out for fun
Driving Cessation Duration<br>(for non-drivers only)	Fixed	Continuous numeric variable for the number of years since last driving a motor vehicle
